# Schematic Assessment of Metabolic Signatures of Non-alcoholic Fatty Liver Disease by Bridging Endocrinology and Internal Medicine: A Precision Therapy-Based Meta-Analysis

**DOI:** 10.7759/cureus.83133

**Published:** 2025-04-28

**Authors:** Syed Muzaffar Abbas, Zeeshan Hussain, Nimra Asghar, Mahnoor Shabbir, Muhammad Armaghan Akhlaq, Hafiz Muhammad Faizan Mughal, Asma Hussain, Abdul Eizad Asif, Ehsan Ul Haq Mzahri

**Affiliations:** 1 Department of General Medicine, Bangor Hospital, Bangor, GBR; 2 Department of Underwater and Hyperbaric Medicine, PNS (Pakistan Navy Station) Shifa Hospital, Karachi, PAK; 3 Department of Diving Medicine, Armed Forces Aero Medical Center, King Abdulaziz Air Base, Dhahran, SAU; 4 Department of Biosciences, COMSATS (Commission on Science and Technology for Sustainable Development in the South) University Islamabad, Islamabad, PAK; 5 Department of General Medicine, Foundation University of Health Sciences, Islamabad, PAK; 6 Department of Otolaryngology, Services Institute of Medical Sciences, Lahore, PAK; 7 Department of Internal Medicine, Khawaja Muhammad Safdar Medical College, Sialkot, PAK; 8 Department of Medicine, Punjab Medical and Dental Clinic, Lahore, PAK; 9 Department of Internal Medicine, Shalamar Medical and Dental College, Lahore, PAK; 10 Department of Health Sciences and Pathology, University of the Punjab, Lahore, PAK; 11 Department of Pathology and Oncology, Forman Christian College, Lahore, PAK; 12 Department of Pathology, University of Indonesia, Jakarta, IDN

**Keywords:** biomarkers, fatty liver, medicine, metabolism, metabolomics, nafld, systematic

## Abstract

Non-alcoholic fatty liver disease (NAFLD) is seen as a health concern globally and is identified via complex interactions of metabolic dysfunctions. Metabolomic and lipidomic profiling has been emerged as a promising tool for non-invasive diagnosis and precision therapy. This systematic review and meta-analysis aimed to assess the affect of metabolic signatures associated with NAFLD progression and their utility in paving path for precision medicine. A comprehensive literature search was conducted in adherence to the guidelines of Preferred Reporting Items for Systematic Reviews and Meta-Analyses (PRISMA) 2020. Appropriate data studies were pooled to check the disease progression using a random effects model. Risk of bias and certainty of evidence were assessed using the Cochrane risk of bias tool, ROBINS-I (“Risk Of Bias In Non-randomized Studies - of Interventions”), and the Grading of Recommendations, Assessment, Development and Evaluation (GRADE) framework respectively. Studies found distinct metabolite patterns especially in amino acids, lipids, and gut-derived metabolites that correlated with the severity of NAFLD. The meta-analysis findings revealed a pooled hazard ratio of 0.98 (95% CI: 0.83-1.15) that indicated that no significant association was found between studies for assessment of metabolic signatures and their link to disease progression. High heterogeneity was observed (I² = 82%). Risk of bias was generally low to moderate, but overall certainty of evidence was rated low to moderate due to inconsistency and imprecision. Metabolic profiling offered valuable insights and discoveries into pathophysiology of NAFLD and stratification. However, high heterogeneity found across studies limited current clinical applicability. Standardized methodologies and longitudinal validation were needed to combine metabolic signatures into precision NAFLD care.

## Introduction and background

Non-alcoholic fatty liver disease (NAFLD) which is now increasingly referred to as metabolic-associated steatotic liver disease (MASLD) has appeared as the most common liver disorder globally which is very prevalent in not only adults but also pediatric populations [1]. This disease is characterized by a condition known as “hepatic steatosis” in the absence of significant alcohol intake [2]. Other than that, NAFLD encompasses a variety of conditions that range from simple steatosis (SS) to non-alcoholic steatohepatitis (NASH), fibrosis, cirrhosis, and hepatocellular carcinoma (HCC) [3]. The complex interactions among metabolic, genetic, and environmental factors in the disease progression of NAFLD demand precise diagnostic and therapeutic strategies [4].

With the emergence of metabolomics and lipidomics researchers are enabled to identify the distinct metabolic signatures that are associated with various stages of NAFLD [5,6]. These signatures are often comprised of amino acids, lipids, organic acids, and bile acids, and reflect upon the underlying pathophysiological processes which also may serve as non-invasive biomarkers to determine disease stage and prognosis [7,8]. Furthermore, the capability to classify the patients into molecular subtypes on the basis of these signatures opens avenues for personalized interventions [9].

This systematic review and meta-analysis aimed to synthesize the results from the available evidence on metabolic profiling in NAFLD. Specifically, this study evaluated how metabolomic and lipidomic signatures had been utilized to identify different stages of disease, to be able to stratify cardiovascular and metabolic risk and to predict the optimum therapeutic response. In addition, this study also conducted a meta-analysis to assess the overall impact of metabolic signatures on the progression of the disease by using hazard ratios (HRs) where available.

## Review

Methodology

This study adhered to the Preferred Reporting Items for Systematic Reviews and Meta-Analyses (PRISMA) 2020 guidelines to ensure transparency and reproducibility. A comprehensive search strategy was put into practice for the identification of studies that investigated metabolic signatures in NAFLD patients. Databases that were employed for this purpose included PubMed, Embase, and Scopus up to March 2025. The keywords that were used to find relevant studies included “NAFLD,” “metabolomics,” “lipidomics,” “biomarkers,” and “precision medicine.” The search included a majority of studies that were published in English and involved human participants.

Inclusion criteria were set for original studies that examined metabolic or lipidomic profiles in NAFLD or MASLD, utilizing the canonical diagnostic criteria (e.g., biopsy, imaging), and presenting quantitative outcomes related to disease characteristics. Exclusion criteria included studies present in languages other than English, case reports, editorials, and reviews. Two independent reviewers first screened titles and abstracts and then performed full-text assessment on studies eligible. Discrepancies or disagreements were resolved either by consensus or consultation with a third reviewer. Primary outcomes of data which was extracted from studies included study design, population characteristics, metabolic markers assessed, analytical techniques, outcomes, and conclusions. Secondary data included any type of statistical values present.

For meta-analysis, pooled HRs (or any of its type) with 95% confidence intervals (CI) along with their lower and upper ranges were calculated using a random effects model to account for study heterogeneity. Heterogeneity was assessed using the I² statistic. Data synthesis and statistical analysis were conducted using a web-based meta-analysis tool. The risk of bias for individual studies was assessed using the appropriate quality assessment tools as per study design. Overall evidence strength was evaluated using the Grading of Recommendations, Assessment, Development and Evaluation (GRADE) framework, where applicable. A PRISMA flowchart was used to document the selection process.

Results

**Figure 1:**
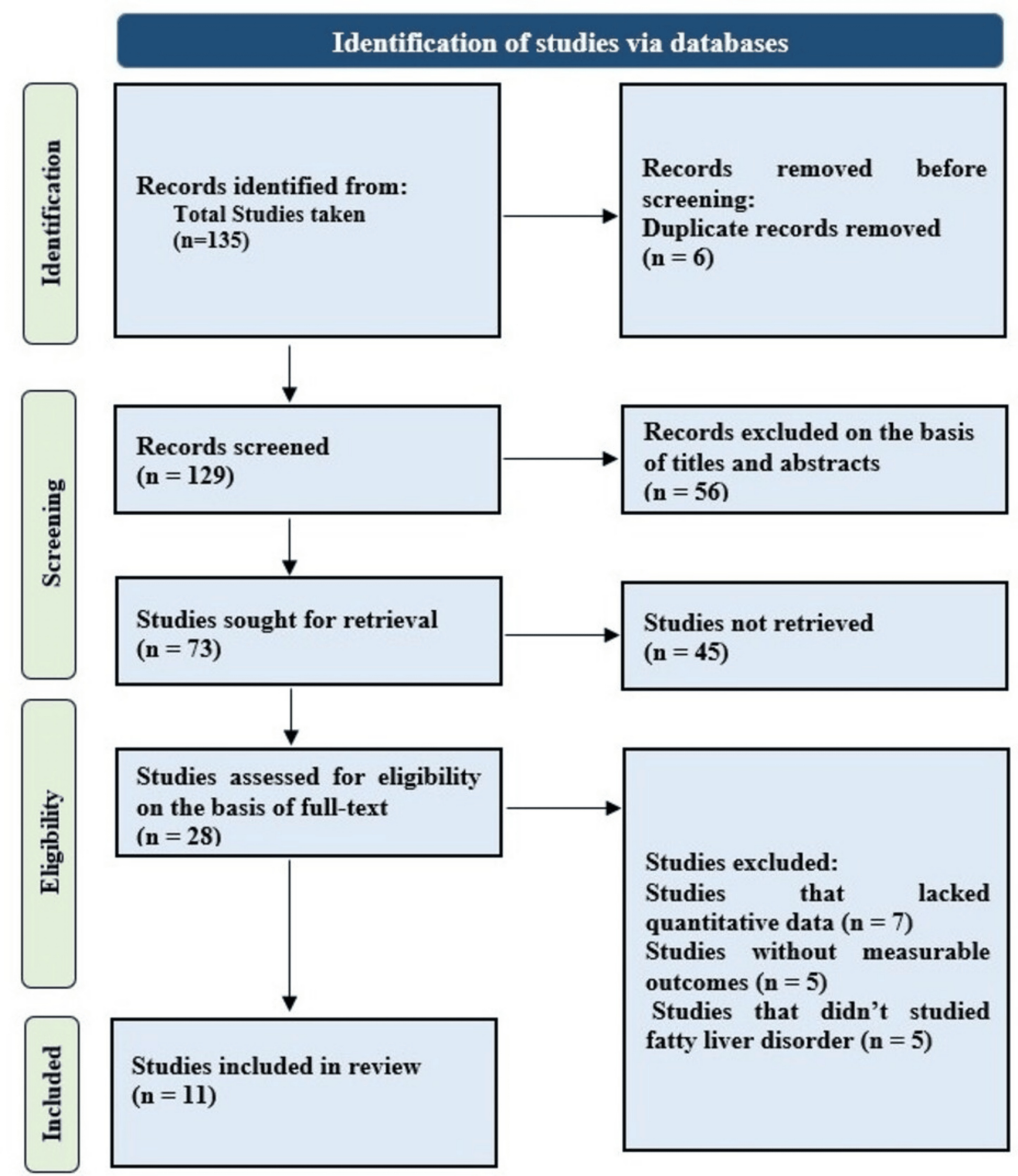
PRISMA flow diagram for study selection process PRISMA: Preferred Reporting Items for Systematic Reviews and Meta-Analyses

A total of 11 studies were included in this systematic review which was comprised of diverse designs of studies such as cross-sectional (n = 7), observational (n = 2), randomized controlled trial (n = 1), and in silico analyses (n = 2). The studies enclosed populations all around the world including Asia, Europe, and America. The studies included both pediatric and adult patients with confirmed NAFLD diagnosis as per established clinical criteria as shown in Figure 1.

**Figure 2:**
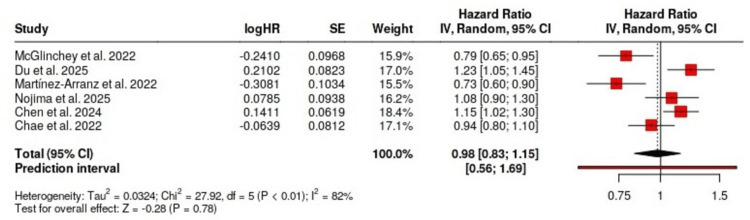
Forest plot showing the pooled hazard ratio (HR) and 95% confidence intervals (CI) for the association between metabolic signatures and NAFLD progression across six included studies NAFLD: non-alcoholic fatty liver disease, logHR: log hazard ration, SE: standard error

The included studies used many metabolomic and lipidomic methods such as ultra-high-performance liquid chromatography quadrupole time-of-flight mass spectrometry (UHPLC-QTOFMS), gas chromatography-mass spectrometry (GC-MS), liquid chromatography-mass spectrometry (LC-MS), and nuclear magnetic resonance (NMR). Both targeted as well as untargeted approaches identified a wide range of metabolic signatures like amino acids (e.g., glutamic acid, branched-chain amino acids {BCAAs}), lipid species (e.g., phosphatidylethanolamines, triglycerides), and gut-derived metabolites (e.g., deoxycholic acid {DCA}). These signatures were seen to be correlated with the stage of disease, inflammation, and grade of fibrosis. Due to the presence of appropriate data type, only six studies contributed to the meta-analysis. A random effects model was used for the yield of pooled HR of 0.98 (95% CI: 0.83-1.15) for disease progression associated with identified metabolic signatures. No significant association was found among studies as heterogeneity was substantial (I² = 82%, p < 0.01) that reflected the differences in analytical techniques, population demographics, and endpoints as shown in the forest plot in Figure 2.

The risk of bias was assessed using the tools appropriate on the basis of study type (e.g., Newcastle-Ottawa Scale for observational studies, ROBINS-I {“Risk of Bias in Non-randomized Studies - of Interventions”} for non-randomized interventions). Most studies showed that the risk of bias was moderate to low, whereas differences in sample size, reporting transparency, and adjustment of confounders reduced overall confidence. The certainty of the evidence was assessed by employing the GRADE framework. Certainty was rated low to moderate because of inconsistency (heterogeneity) and imprecision in effect estimates as well as indirectness of outcomes. Despite promising individual findings there was inconsistency across studies that limited strong conclusions regarding the predictive power of metabolic signatures. Overall, the results support the potential clinical applications but further standardization and prospective research are needed to validate findings. Systematic evaluation of characteristics of individual selected studies can be seen in Table 1.

**Table 1:** Systematic evaluation of characteristics of individual selected studies AAMRG: amino acid metabolism-related genes, AAs: amino acids, AUC: area under the curve, AUROC: area under the receiver operating characteristic curve, BCAA: branched-chain amino acids, CVD: cardiovascular disease, DAPC: discriminant analysis of principal components, DCA: deoxycholic acid, DEG: differentially expressed genes, FFA: free fatty acids, GC-MS: gas chromatography-mass spectrometry, GC-QTOFMS: gas chromatography quadrupole time-of-flight mass spectrometry, GSEA: gene set enrichment analysis, GSVA: gene set variation analysis, LASSO: least absolute shrinkage and selection operator, LC-MS: liquid chromatography-mass spectrometry, LEfSe: linear discriminant analysis effect size, LGIMD: likely genetic inherited metabolic disorder, LMRG: likely metabolic-related genes, MASLD: metabolic dysfunction-associated steatotic liver disease, ML: machine learning, NAFL: non-alcoholic fatty liver, NAFLD: non-alcoholic fatty liver disease, NASH: non-alcoholic steatohepatitis, NL: normal liver, NMR: nuclear magnetic resonance, PC: phosphatidylcholine, PCA: principal component analysis, PE: phosphatidylethanolamine, PERMANOVA: permutational multivariate analysis of variance, qPCR: quantitative polymerase chain reaction, qRT-PCR: quantitative reverse transcription polymerase chain reaction, RC: reference control, RF: random forest, ROC: receiver operating characteristic, SM: sphingomyelin, SS: simple steatosis, SVM: support vector machine, TG: triglycerides, UHPLC-MS: ultra-high-performance liquid chromatography-mass spectrometry, UHPLC-QTOFMS: UHPLC-quadrupole time-of-flight mass spectrometry, UPLC-MS/MS: ultra-performance liquid chromatography tandem mass spectrometry, VLDL-TG: very low-density lipoprotein triglycerides, WB: western blot, WGCNA: weighted gene co-expression network analysis

Authors and year	Study design and population	Metabolic signatures analyzed	Methods used	Key findings	Conclusions	Risk assessment
McGlinchey et al., 2022 [10]	Cross-sectional; 627 biopsy-proven NAFLD patients (European NAFLD Registry)	176 serum lipids, 36 polar metabolites	UHPLC-QTOFMS, GC-QTOFMS; univariate, multivariate & machine learning	Distinct metabolic profiles associated with steatosis, NASH, and fibrosis; transition point at F2–F3	Metabolomics/lipidomics track NAFLD progression; support early intervention	Low
Du et al., 2025 [11]	Observational; 36 children (microbiota), 25 (metabolomics); MASLD, obesity, and controls (ages 6–16)	310 fecal metabolites; gut microbiota	UPLC-MS/MS, 16S rDNA, LEfSe, PCA, PERMANOVA, qPCR	Ruminococcus torques & DCA associated with MASLD; low gut microbial diversity	Microbiota–metabolite axis offers diagnostic & therapeutic targets for pediatric MASLD	Moderate
Martínez-Arranz et al., 2022 [12]	Cross-sectional; 1,154 biopsy-proven NAFLD adults (EU, US, Chile)	Serum metabolome, VLDL-TG secretion, lipoprotein profiles	Serum metabolomics, Framingham score, genetic risk markers	Identified 3 NAFLD subtypes with distinct CVD risk profiles	NAFLD metabolic subtypes stratify CVD risk independent of liver histology	Low
Nojima et al., 2025 [13]	Cross-sectional; 3,733 Japanese adults (excluded alcohol users, diabetics)	114 serum metabolites (AAs, organic acids)	GC-MS, LASSO, ROC, Pearson, t-test	Glutamic acid upregulated in NAFLD; model AUC = 0.866; “pre-NAFLD” detected	Metabolomics aids early NAFLD diagnosis, especially in lean individuals	Low
Chen et al., 2024 [14]	Cross-sectional; 250 obese Chinese adults with biopsy-confirmed NAFLD stages	263 metabolites + 550 lipids (TG, PC, PE, etc.)	Untargeted LC-MS, WGCNA, MaAsLin2, logistic regression	Lipidomic shifts from SS to NASH; BCAAs, TGs, and PEs key differentiators	Multi-omics facilitates staging biomarkers and noninvasive NASH prediction	Low
Chae et al., 2022 [15]	Cross-sectional; 165 Korean children/adolescents grouped by BMI & ultrasound	342 plasma metabolites (AAs, lipids, acylcarnitines)	Targeted metabolomics (AbsoluteIDQ™), ML (ElasticNet, RF, XGBoost)	18 NAFLD-specific metabolites; AUC ~0.95; altered BCAA and lipid metabolism	Accurate pediatric NAFLD classification using combined omics and ML tools	Moderate
Calabrese et al., 2022 [16]	RCT; 109 NAFLD patients randomized to 6 lifestyle arms	Gut microbiota taxa; predicted metabolic pathways	16S rRNA sequencing, DAPC, MaAsLin2, Picrust2	LGIMD + aerobic activity reshaped gut microbiota, reduced steatosis	Gut–liver axis responds to lifestyle; combined diet and exercise most effective	Moderate
Fotakis et al., 2024 [17]	Case–control; 223 Greek adults (89 MASLD, 134 controls)	Circulating metabolite ratios (e.g. alanine/formic acid)	Serum NMR, PCA, AUROC, MetaboAnalyst	Elevated alanine/formic acid & leucine/formic acid in poor-lifestyle MASLD	Metabolite ratios may serve as lifestyle-linked biomarkers of MASLD risk	Moderate
Liu et al., 2023 [18]	In silico study, 142 training and 57 test individuals with NAFLD vs healthy controls	LMRGs and immune infiltration signatures	WGCNA, GSVA, consensus clustering, ML (RF, SVM)	2 NAFLD subtypes identified; 5-gene predictive model built	LMRG subtypes link metabolic and immune features; model supports risk stratification	Low
Li J et al., 2025 [19]	In silico study of 2256 DEGs from NAFLD datasets (GSE89632, GSE135251); validated in NAFLD mouse model	AAMRGs: CYP2U1, GGT1, PLA2G1B, GPX2, PTGS1	DEG analysis, LASSO, logistic regression, qRT-PCR, WB, ROC, GSEA	5-gene panel (AUC > 0.7) diagnostic of NAFLD; linked to oxidative stress & inflammation	AAM pathway genes are promising diagnostic and therapeutic targets in NAFLD	Low
Mowry et al., 2021 [20]	Cross-sectional; 37 liver transplant recipients (NAFL, NASH, NL, RC groups)	434 plasma metabolites (TG, FFA, PC, SM, AAs)	UHPLC-MS, PCA, hierarchical clustering, Wilcoxon test	14 metabolites differentiated NAFLD from controls; 16 separated NAFL vs NASH	Noninvasive metabolic profiling may monitor NAFLD recurrence post-LT	High

Discussion

This study evaluated the context in which metabolomic and lipidomic signatures contributed to NAFLD and their potential for directing precision medicine approaches. The results highlighted the heterogeneous and multifactorial in nature traits of NAFLD as a whole, and metabolic signals show that there is great potential for patient classification and disease monitoring. Most studies focused on the fact that targeted and untargeted metabolomics differ in their applications to different stages of NAFLD [21]. For example, both glutamic acid and BCAAs increased uniformly with the advancing disease and likely had some role in hepatocellular damage and inflammation [22]. Changes were reported in lipidomic studies of triglyceride (TG), phosphatidylcholine (PC) as well as phosphatidylethanolamine (PE) profiles where the transition from SS to NASH was particularly noteworthy. Such changes correspond with derangements of lipid metabolism as well as mitochondrial impairment and oxidative stress, all components of NAFLD pathology [23]. Apart from this, there have been discoveries about new models that diagnose diseases through the integration of multi-omics data with machine learning [24]. For example, there are those models that obtained classifiers with accuracies near those of indicators based on metabolite panel or gene-metabolite associations (AUC value nearly 0.95). This represents the application of computational tools to improve the diagnosis of metabolic signatures, among others [25].

Key metabolites which are derived from gut micromedia, such as deoxycholic acid (DCA) and short-chain fatty acids have been figured mostly in pediatric studies and in fewer adult studies. These suggested that the gut-liver axis are potentially modifiable target in NAFLD [26]. One of the experimental study results indicated that lifestyle changes, including low glycemic diets and aerobic exercises, can change a person's gut microbiota and some of the associated metabolic pathways, rendering therapeutic access [27]. The combined HR value from six studies totaling 0.98 with a 95% confidence interval ranging between 0.83-1.15 showed no significant relationship between studies as high heterogeneity (I² = 82%) limited interpretability. The research variability arose from multiple factors including study design and analytical platforms together with participants (children vs adults) and the endpoints examined. Researchers focused their efforts on either diagnosing or modeling prognosis in their studies.

Standardization of platforms for metabolomics and analytical pipelines is necessary for comparability within studies. Future efforts should focus on homogenization of methodologies and validation of candidate biomarkers among diverse populations to enable clinical translation. Larger, longitudinal cohorts are required to validate the findings and to assess the predictive capabilities over time.

## Conclusions

This review highlighted the potential role of metabolomic and lipidomic signatures in identifying the characteristics of NAFLD and informing individualized care. While current evidence demonstrated that there was a diagnostic potential, substantial heterogeneity, and methodological variability prevented definitive conclusions. The meta-analysis did not find a significant association between studies that underscored the need for standardization and larger prospective studies.

Nonetheless, combining metabolic data with the clinical and molecular parameters might enhance the early diagnosis and risk classification. Future research should focus on the validation of biomarkers across a variety of populations and harmonizing the analytical approaches to enable their translation into routine clinical practice.
